# Surgical Option in the Management of a Giant Vesical Calculus Post Augmentation Cystoplasty

**DOI:** 10.7759/cureus.61037

**Published:** 2024-05-25

**Authors:** Abdulhkam Aljarbou, Mohammed Alturki, Suliman Alnamlah, Mutasim Alkhalifah, Faisal Alturki

**Affiliations:** 1 Urology, King Saud Medical City, Riyadh, SAU; 2 Urology, King Fahad Medical City, Riyadh, SAU

**Keywords:** open cystolithotomy, urinary bladder, ileocystoplasty, vesical calculus, bladder augmentation

## Abstract

We describe a rare instance of enormous, calcified stone development in an enlarged urinary bladder. The patient was a 24-year-old female suffering from the development of vesical calculus as a result of complicated bladder augmentation. She had a small bladder capacity and had undergone augmentation ileocystoplasty in childhood. The abdomen was examined using X-ray and computerized tomography, which revealed a significantly huge urinary bladder stone. An open cystolithotomy was performed and a giant vesicle calculus was extracted along with another small stone. Although stone development is a typical side effect following augmented bladder, it may be avoided with frequent bladder irrigation and attentive aftercare.

## Introduction

Vesicle calculus, or bladder stones, is a medical condition in which mineral deposits accumulate in the bladder, causing pain and discomfort. While the condition can occur due to various factors, one of the less well-known causes is complicated bladder augmentation. Bladder augmentation, also known as augmentation cystoplasty, is a surgical procedure used to increase the size of the bladder by grafting tissue from another part of the body onto the bladder wall. Such a procedure is commonly performed to treat patients with conditions such as myelomeningocele, spina bifida, or spinal cord injuries, who may experience urinary incontinence or retention due to a small bladder size [1].

While bladder augmentation can improve bladder function and quality of life for these patients, it can also lead to complications, including the development of vesical calculus [1]. The changes in the bladder environment caused by the surgery, including decreased bladder capacity and altered urinary pH levels, increase the risk of mineral buildup within the bladder, forming bladder stones. Patients may experience pain or discomfort during urination, frequent urination, blood in the urine, and difficulty passing urine. If left untreated, bladder stones can cause further complications, including obstruction of the urinary tract and kidney damage.

Diagnosing vesical calculus can be done through imaging tests such as ultrasound, X-rays, or CT scans. Treatment options depend on the size and location of the bladder stones. Some stones are small, while those that weigh greater than or equal to 100 g are referred to as big stones [2]. In this current case study, we present a massive vesical stone and a small stone, which developed in the bladder after the augmentation of the urinary bladder.

## Case presentation

**Figure 1:**
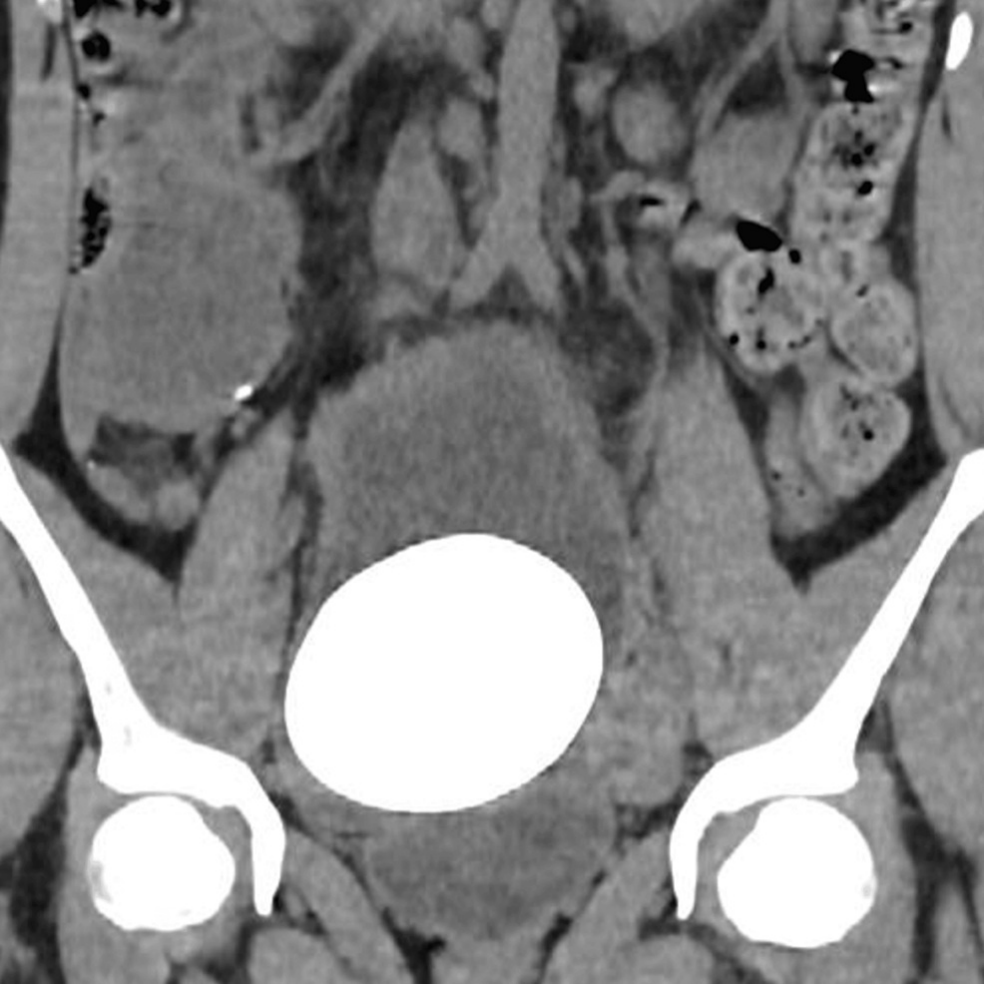
Computer tomography showing huge bladder stone in coronal view

A 24-year-old female patient, a known case of neurogenic bladder due to myelomeningocele, bilateral grade V vesicoureteral reflux, chronic kidney disease, post ileocystoplasty 20 years ago, and on intermitted catheterization, presented with recurrent urinary tract infections (UTI). During the regular examination by CT, the patient was found to have a massive stone burden in the urinary bladder (Figure 1). The urine analysis reported pus cells along with leukocytes and bacterial cultures were positive for *Klebsiella pneumoniae,* which was susceptible to antibiotics. Following the management of the bladder infection, the patient was scheduled for surgical interventions. 

**Figure 2:**
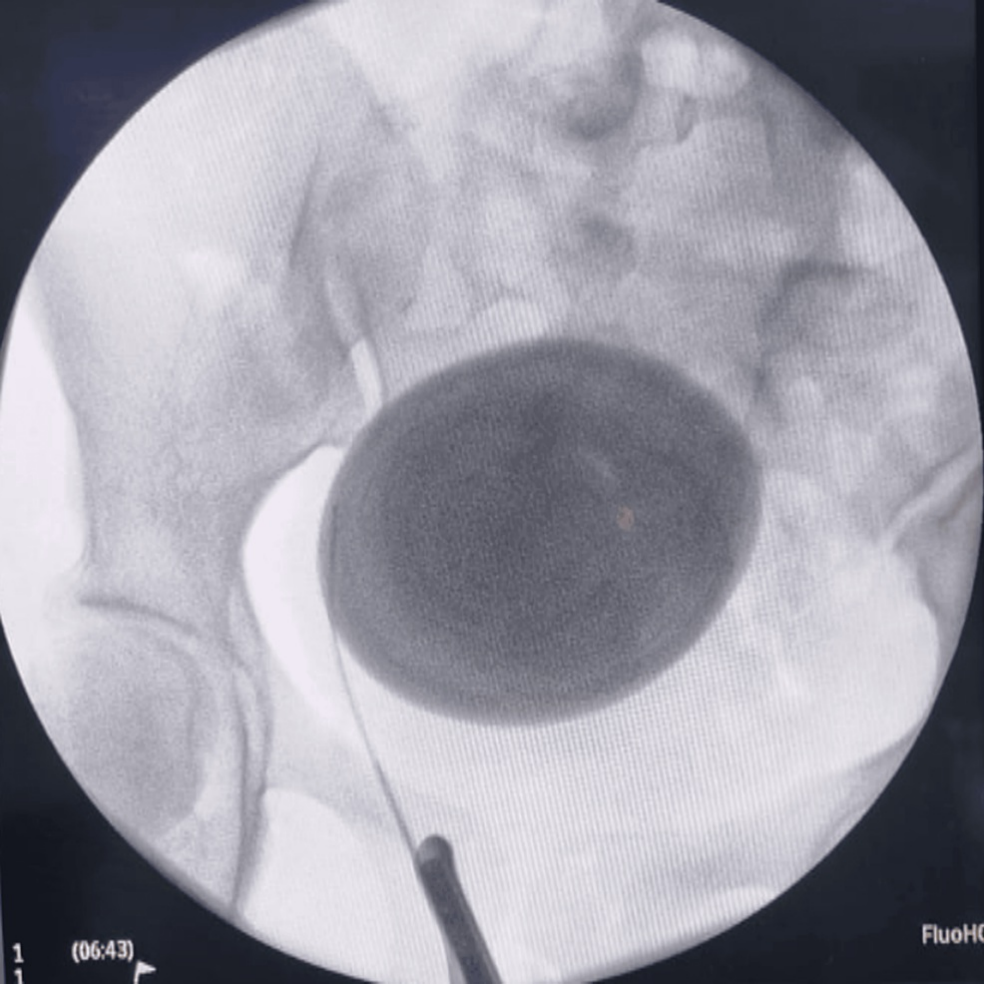
X-ray fluoroscopy with right retrograde pyelogram showing no stone in right ureter

**Figure 3:**
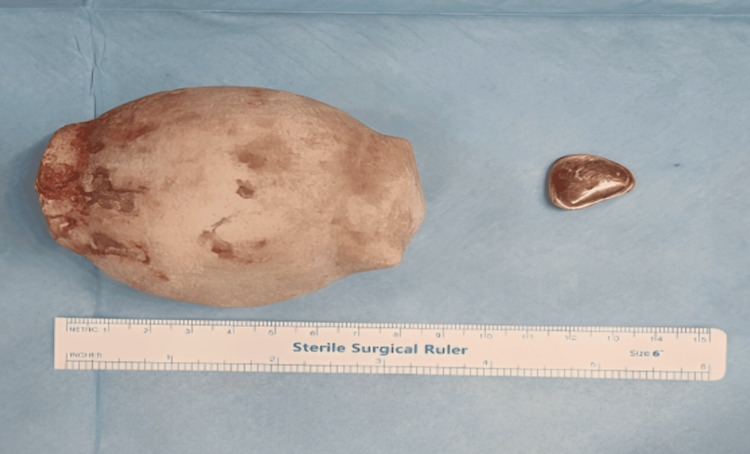
One enlarged bladder stone removed through open cystolithotomy

The patient underwent a cystoscopy and right retrograde pyelogram to rule out any right distal ureteric stone (Figure 2). This was followed by open cystolithotomy under general anesthesia, resulting in the removal of a giant stone, oval in shape measuring 9 cm in length and 8 cm in width, along with a small stone (Figure 3).

A silicone Foley catheter was inserted and the patient was shifted to the general ward for monitoring. Postoperatively, the patient was covered by IV antibiotics and analgesia. On postoperative day 5, the patient was discharged home. A cryptogram was performed on postoperative day 10 and revealed no urine extravasation. The Foley catheter was removed and the patient resumed clean intermittent catheterization (CIC). 

## Discussion

Vesical calculus, commonly known as bladder stones, is a condition characterized by the formation of solid crystalline deposits within the urinary bladder. It is a common condition that can occur in individuals of all ages and genders but mostly afflicts males in their middle and later years. In some cases, the development of large bladder stones can be attributed to complicated bladder augmentation, a surgical procedure that enlarges the bladder. The present case involved a large vesical calculus that developed due to complicated bladder augmentation.

Complicated bladder augmentation is a procedure that enlarges the bladder to improve bladder function in patients with bladder dysfunction. It is often used in patients with conditions such as spina bifida, bladder exstrophy, and neurogenic bladder. The procedure involves the use of bowel tissue to increase the size of the bladder. However, this can lead to changes in the composition of urine, making it more conducive to the formation of bladder stones [3]. In the present case, the patient had a history of complicated bladder augmentation using bowel tissue. Over time, the patient began to experience symptoms such as pain and difficulty urinating. Upon further examination, it was discovered that the patient had developed a large bladder stone. The development of such a large bladder stone can cause significant discomfort and complications for the patient. The stone can obstruct the flow of urine, leading to urinary retention, infection, and other complications. Additionally, the size of the stone can cause damage to the bladder wall, leading to bleeding and inflammation [4].

Open cystolithotomy is an effective treatment for large stone burdens, as it allows for the complete removal of stones and enables examination of the bladder for any associated abnormalities. Analytical testing revealed that the crystals were of the struvite kind. Struvite stones are commonly associated with UTIs caused by urease-producing bacteria, such as *Proteus* and *Klebsiella* species. The presence of these stones indicates chronic infection and should prompt further evaluation for underlying anatomical abnormalities that may predispose to recurrent infections. The smaller pieces are removed from the bladder using a laser or ultrasonic device. After the surgical removal of the bladder stone, the patient experienced significant relief from their symptoms. However, there are risks associated with the surgical removal of a large bladder stone, including bleeding, infection, and injury to the bladder. The use of a cystoscope during the surgical procedure can also cause trauma to the urethra, leading to scarring and other complications. Regular follow-up appointments with a healthcare provider can also help to identify the development of bladder stones at an early stage [5].

## Conclusions

The development of a large vesical calculus is a serious complication of bladder augmentation. Changes in the composition of urine due to the use of bowel tissue in the procedure can contribute to the formation of bladder stones. The treatment of large bladder stones typically involves surgical removal, which carries risks of complications. Preventive measures such as maintaining good bladder hygiene and regular monitoring of urine composition can help to reduce the risk of developing bladder stones in patients with complicated bladder augmentation.
